# The effect of nitrates in patients with coronary artery disease across different left ventricular ejection fractions

**DOI:** 10.3389/fcvm.2026.1662112

**Published:** 2026-02-19

**Authors:** Hui Liu, Wei Liu, Yalin Cao, Zongzhuang Li, Piao Zheng

**Affiliations:** 1 Department of Cardiology, Guizhou Provincial People’s Hospital, Guiyang, Guizhou, China; 2 Department of Cardiology, Zunyi Medical University, Zunyi, Guizhou, China

**Keywords:** all-cause mortality, coronary artery disease, left ventricular ejection fraction, major adverse cardiovascular events, nitrates

## Abstract

**Background:**

Long-acting nitrates are widely prescribed in coronary artery disease (CAD), yet their association with long-term outcomes remains controversial. Whether left ventricular ejection fraction (LVEF) modifies this relationship has not been well characterized.

**Methods:**

We conducted a single-center retrospective cohort study using the Guizhou Provincial People's Hospital CAD database. Adults (≥18 years) who underwent coronary angiography between July 2012 and September 2016 and met angiographic criteria for CAD (≥50% stenosis in ≥1 proximal epicardial coronary artery) were eligible if LVEF and discharge medications were available. Patients were stratified by LVEF (<45%, 45%–55%, >55%) and by discharge prescription of any nitrate (mononitrate or dinitrate). The primary endpoint was all-cause death. The secondary endpoint was major adverse cardiovascular events (MACE), defined as a composite of cardiovascular death, non-fatal myocardial infarction, and non-fatal stroke. Kaplan–Meier methods and multivariable Cox regression were used to evaluate associations.

**Results:**

Among 2,404 patients followed for 27.2 ± 13.5 months, 1,153 (48.0%) were discharged on nitrates. In the overall cohort, discharge on nitrates was not associated with all-cause death or MACE. In contrast, among patients with LVEF < 45%, nitrates at discharge were linked to higher cumulative incidences of all-cause death and MACE (log-rank *P* = 0.024 and 0.029, respectively) and remained independently associated after adjustment [all-cause death: hazard ratio (HR) 2.14, 95% confidence interval (CI) 1.15–3.96; MACE: HR 1.91, 95% CI 1.07–3.43]. No significant associations were observed in the LVEF 45%–55% or >55% strata.

**Conclusion:**

In this CAD cohort, nitrates were commonly prescribed at discharge. An adverse association with long-term outcomes was confined to patients with reduced LVEF, supporting cautious use in this subgroup and highlighting the need for prospective confirmation.

## 1 Introduction

Despite more than 140 years of clinical use, the long-term prognostic impact of nitrates remains uncertain (1). Nitrates continue to be among the most commonly prescribed medications for patients with coronary artery disease (CAD). According to current guidelines, long-acting nitrates may be used for angina prevention or symptom relief in patients who remain symptomatic despite treatment with aspirin, statins, β-blockers, or calcium channel blockers (2). In addition CAD, nitrates also have been recommended in the treatment of congestive heart failure (3). However, controversy surrounding nitrate use has persisted.

Several studies have suggested that long-term nitrate therapy may be associated with increased adverse clinical events in patients with CAD. Nevertheless, few studies have explicitly examined whether baseline left ventricular ejection fraction (LVEF) modifies this association (4–6). In some studies involving patients with reduced LVEF, nitrate treatment has demonstrated convincing benefits in terms of remodeling and exercise tolerance. In the case of African-American patients with LVEF ≤ 45%, nitrate treatment was even associated with reduced mortality (7, 8). However, approximately half of the patients enrolled in these studies did not have CAD. Therefore, evidence regarding the impact of nitrate therapy on patients with CAD across different levels of LVEF remains limited.

Discharge medications, as a critical component of the discharge summary, play a very important role in patient safety and continuity of care (9). The purpose of this study was therefore to analyze and compare if CAD patients discharged on nitrates experienced different prognoses across different levels of LVEF.

## 2 Methods

### 2.1 Data collection

We retrospectively selected patients diagnosed with coronary heart disease from the Department of Cardiology at Guizhou Provincial People's Hospital between July 2012 and August 2016. Eligible patients were aged 18 years or older and had undergone coronary angiography. CAD was defined as angiographic evidence of ≥50% diameter stenosis in at least one major epicardial coronary artery (left main, LAD, LCx, or RCA), regardless of segment location (proximal, mid, or distal) (10, 11). Detail definitions of acute coronary syndrome and stable angina have been previously described (12). The hospital database collects information about medical history, cardiovascular risk factor, previous medication, clinical characteristics, laboratory variables, echocardiographic results, angiographic findings, revascularization procedures, medication on discharge, and follow-up data.

### 2.2 Study population

A total of 3,714 patients diagnosed with coronary heart disease in the cardiology department at Guizhou Provincial People's Hospital between July 2012 and August 2016 were retrospectively selected. Eligible patients had ≥50% stenosis in at least one major epicardial vessel, irrespective of lesion segment. The analyses in this study were restricted to patients with detailed discharge medication records and documented LVEF. Patients discharged on nitrates were defined as those prescribed any formulation of nitrates (i.e., mononitrate or dinitrate) at discharge. Typically, patients are discharged with a 2-week supply or more in this hospital. The following groups of patients were excluded from this study: patients who died during hospitalization; patients lost to follow-up; patients with incomplete follow-ups; patients who did not undergo at least one echocardiographic examination; and patients with missing data on discharged medicines. Guideline-directed medicine treatment was defined *a priori* as evidence-based pharmacologic therapy recommended for chronic coronary disease, including antiplatelet therapy and statins for secondary prevention, as well as beta-blockers and angiotensin-converting enzyme inhibitors or angiotensin receptor blockers when clinically indicated (13). Antianginal agents, including calcium channel blockers and long-acting nitrates, were prescribed for symptom control at the treating physicians’ discretion, consistent with contemporary guideline recommendations (13).

### 2.3 Follow-up

Follow-up was completed primarily through telephone interviews. All interviewers were trained professionally prior to conducting interviews. A predefined questionnaire was used to collect the follow-up information. If patients could not be contacted or declined telephone follow-up, alternative methods—including chart review, clinical attendance records, or letter correspondence—were used.

### 2.4 Endpoints and definitions

The primary endpoint of this study was all-cause death. The secondary endpoint was major adverse cardiovascular events (MACE) [defined as the composite of cardiovascular death (CV death), non-fatal myocardial infarction (MI), and non-fatal stroke]. All events were adjudicated by two independent members.

### 2.5 Statistical analysis

Patients were divided into three groups according to LVEF status. Group 1 was LVEF less than 45%, group 2 was LVEF between 45% and 55%, and group 3 was LVEF more than 55% (14). This categorization was selected *a priori* to reflect commonly used echocardiographic grading of systolic function, in which LVEF >55% is generally considered normal, 45%–54% is considered mildly reduced, and values below this range indicate at least moderate systolic dysfunction. In addition, contemporary evidence in coronary artery disease supports 55% as a clinically relevant prognostic threshold, with higher mortality observed below an LVEF of approximately 55% in a large CAD cohort (15). To facilitate comparison with ESC heart failure nomenclature, we performed a sensitivity analysis using ESC LVEF categories [Heart failure with reduced ejection fraction(HFrEF) ≤ 40%, Heart failure with mildly reduced ejection fraction (HFmrEF) 41%–49%, and Heart failure with preserved ejection fraction (HFpEF) ≥ 50%]. In each group, patients were further stratified into two subgroups based on whether they were discharged with or without nitrates. The prescription of nitrates was at the discretion of the doctor. Data were compared between patients discharged with and without nitrates in each group. Continuous variables are expressed as mean ± standard deviation (SD), and categorical variables are reported as absolute value [percentages (%)]. Continuous and categorical variables were compared using analysis of variance and chi-square tests, respectively. Kaplan–Meier analyses were performed to estimate the cumulative incidences of primary and secondary outcomes, stratified by discharged with or without nitrates. Cox proportional hazards regression analysis was performed to examine factors associated with outcomes. The effect size was hazard ratios (HRs) and its corresponding 95% conﬁdence intervals (95% CI). In multivariate Cox proportional hazards regression analysis, clinical risk factors included age, gender, diabetes, hypertension, and smoking. Additional covariates were selected based upon univariate analyses or baseline comparisons. Variables entered the analyses if the univariate analysis showed *p* ≤ 0.2 or if baseline comparisons suggested signiﬁcant differences between subgroups. All tests were two-sided, and *p* ≤ 0.05 was considered statistically signiﬁcant. All analyses were performed using SPSS software (Version 19.0). Given the retrospective nature of the study, the sample size was determined by the available data in the database. A *post-hoc* power analysis was performed to estimate the statistical power of the LVEF subgroups. We calculated the power to detect a clinically meaningful HR of 1.5–2.0 for all-cause death, assuming a two-sided alpha of 0.05 and using observed event rates in the control groups (PASS 15.0, NCSS, LLC).

## 3 Results

**Figure 1:**
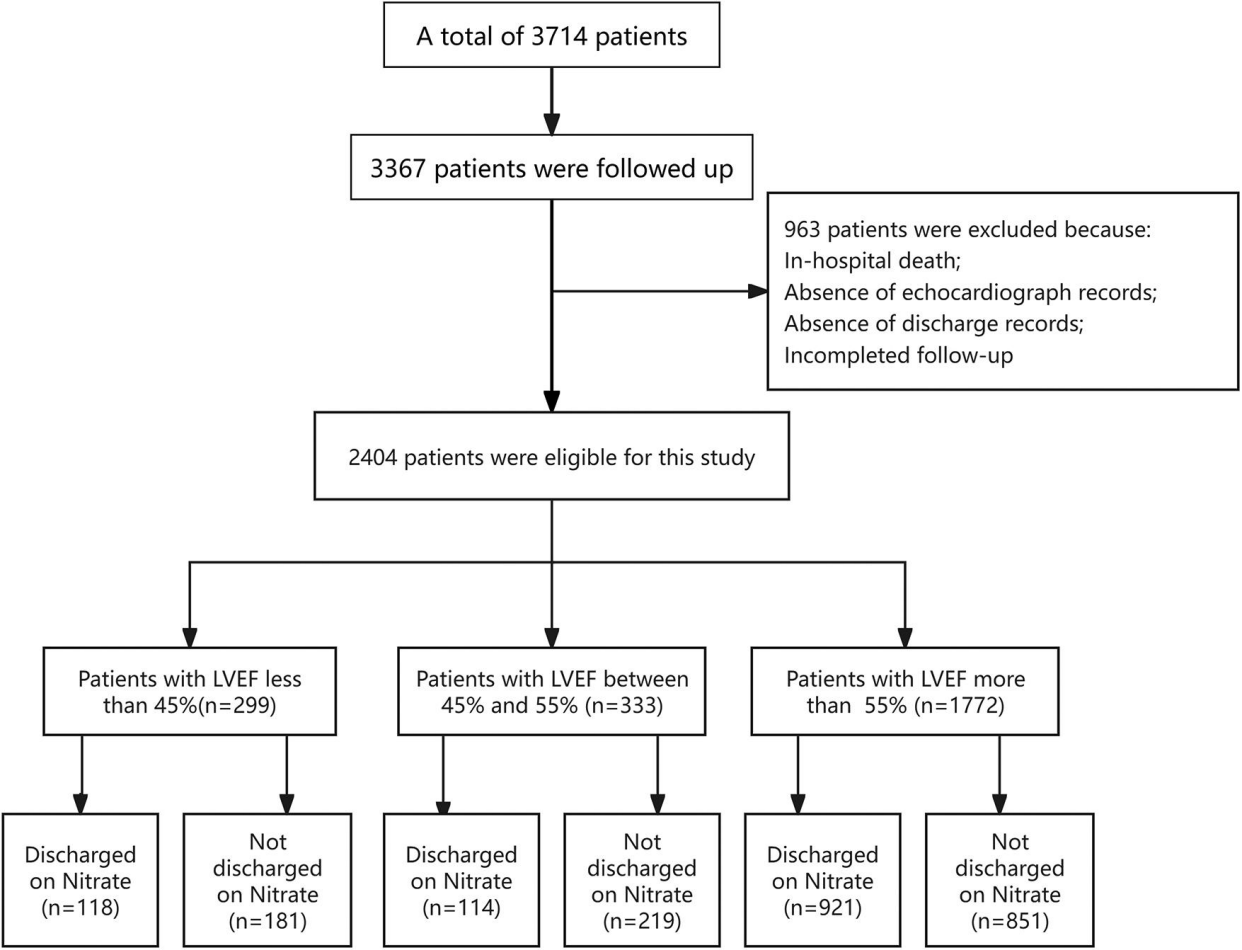
Patient screening and disposition.

A total of 3,714 patients diagnosed with coronary heart disease by coronary angiography in the cardiology department at Guizhou Provincial People's Hospital between July 2012 and August 2016 were retrospectively selected. Of these, 3,367 patients (90.7%) completed follow-up, while 347 patients (9.3%) were lost to follow-up. The mean duration of follow-up was 27.2 ± 13.5 months. The follow-up flowchart is presented in Figure 1. Among the 3,367 patients, another 963 patients were excluded from this study because of in-hospital death, incomplete follow-up, absent echocardiograph records, or missing discharge records. Finally, 2,404 patients were eligible for this study. Of these, 1,153 patients (48%) were discharged on nitrates and 1,251 patients (52%) were not discharged on nitrates. Classifying these patients into three groups based upon LVEF, 299 patients had LVEF < 45%, 333 LVEF between 45% to 55%, and 1,772 LVEF > 55%.

The detailed baseline characteristics of the study population are shown in Table 1. Based on these sample sizes, a *post-hoc* power analysis indicated that the statistical power was sufficient (>80%) to detect clinically relevant hazard ratios in the LVEF < 45% and >55% subgroups, which comprised the majority of the study population. Compared with patients not discharged on nitrates, patients discharged on nitrates had similar distributions of sex, acute coronary syndrome, diabetes, body mass index, and LDL-C. However, patients with nitrates at discharge were older, appeared to have more hypertension, were less likely to have a history of myocardial infarction, and had fewer current smokers. Moreover, these patients seemed to have higher LVEF and serum creatinine, and fewer implanted stents during the index procedures. Importantly, patients discharged on nitrates seemed to take aspirin, statin, angiotensin-converting enzyme inhibitor (ACEI)/angiotensin receptor blocker (ARB), β-blockers, and calcium channel blocker (CCB) before admission. However, patients discharged on nitrates received comparable rates of aspirin, clopidogrel, and ACEI/ARB, but were prescribed fewer statin and more CCB and β-blockers.

**Table 1:** Baseline characteristics by level of left ventricular ejection fraction.

Variables	Total (*N* = 2,404)		*P*	LVEF < 45% (*N* = 299)		*P*	45% ≤ LVEF ≤ 55% (*N* = 333)		*P*	LVEF > 55% (*N* = 1,772)		*P*
	With nitrates	Without nitrates		With nitrates	Without nitrates		With nitrates	Without nitrates		With nitrates	Without nitrates	
No. of patients *n* (%)	1,153 (48.0)	1,251 (52.0)	118 (39.5)	181 (60.5)	114 (34.2)	219 (65.8)	921 (52.0)	851 (48.0)				
Male, *n* (%)	905 (78.5)	1,007 (80.5)	0.224	97 (82.2)	159 (87.8)	0.174	92 (80.7)	186 (84.9)	0.324	71.6 (77.7)	662 (77.8)	0.980
Age, years	65.3 (10.1)	63.8 (10.7)	0.000	65.4 (10.0)	64.8 (10.8)	0.630	63.2 (11.7)	62.1 (11.1)	0.392	65.5 (9.8)	64.0 (10.6)	0.002
ACS, *n* (%)	829 (71.9)	862 (69.0)	0.118	82 (69.5)	129 (71.3)	0.741	85 (74.6)	157 (71.7)	0.577	662 (71.9)	577 (67.8)	0.062
History of MI, *n* (%)	266 (23.1)	404 (32.3)	0.000	55 (46.6)	92 (50.8)	0.476	41 (36.0)	100 (45.7)	0.089	170 (18.5)	212 (24.9)	0.001
History of PCI, *n* (%)	139 (12.1)	147 (11.8)	0.818	15 (12.7)	22 (12.2)	0.886	12 (10.5)	20 (9.1)	0.682	112 (12.2)	105 (12.3)	0.909
Hypertension, *n* (%).	684 (59.3)	656 (52.4)	0.001	68 (57.6)	73 (40.3)	0.003	59 (51.8)	101 (46.1)	0.329	557 (60.5)	482 (56.6)	0.101
Diabetes milletus, *n* (%)	257 (22.3)	278 (22.2)	0.968	32 (27.1)	38 (21.0)	0.222	31 (27.2)	50 (22.8)	0.379	194 (21.1)	190 (22.3)	0.519
Current Smoker, *n* (%)	300 (26.0)	397 (31.7)	0.002	33 (28.0)	64 (35.4)	0.182	33 (28.9)	80 (36.5)	0.166	234 (25.4)	253 (29.7)	0.042
BMI, kg/m^2^	24.3 (3.3)	24.1 (3.0)	0.088	23.9 (3.3)	23.8 (2.7)	0.728	24.6 (3.9)	24.1 (2.7)	0.203	24.4 (3.2)	24.2 (3.1)	0.275
Waist-Hip ratio	0.96 (0.66)	0.96 (0.69)	0.533	0.96 (0.53)	0.95 (0.49)	0.167	0.96 (0.48)	0.96 (0.52)	0.336	0.96 (0.69)	0.96 (0.76)	0.986
LVEF, %	61.7 (11.1)	58.8 (11.8)	0.000	36.6 (6.2)	36.7 (6.1)	0.890	50.1 (3.2)	50.3 (3.1)	0.717	66.3 (5.4)	65.6 (5.1)	0.009
Serum creatinine, µmol/L	96.5 (62.3)	91.9 (41.5)	0.030	98.8 (32.4)	98.6 (43.1)	0.956	99.6 (81.6)	97.0 (66.3)	0.752	95.9 (62.5)	89.1 (31.5)	0.004
Glucose, mmol/L	6.7 (2.9)	7.2 (3.5)	0.000	7.4 (3.9)	7.4 (3.6)	0.947	7.6 (3.6)	8.3 (4.2)	0.126	6.5 (2.6)	6.9 (3.3)	0.007
Total cholesterol, mmol/L	4.1 (1.1)	4.1 (1.1)	0.539	4.0 (1.0)	4.1 (1.1)	0.672	4.1 (1.2)	4.2 (1.4)	0.288	4.1 (1.1)	4.1 (1.0)	0.904
Triglyceride, mmol/L	1.8 (1.1)	1.7 (1.2)	0.462	1.6 (0.8)	1.6 (1.0)	0.674	1.7 (0.9)	1.9 (1.6)	0.235	1.8 (1.1)	1.7 (1.1)	0.131
HDL-C, mmol/L	1.1 (0.4)	1.2 (0.4)	0.458	1.1 (0.3)	1.1 (0.3)	0.824	1.1 (0.3)	1.1 (0.3)	0.358	1.2 (0.4)	1.2 (0.4)	0.349
LDL-C, mmol/L	2.4 (0.9)	2.4 (0.9)	0.314	2.4 (0.9)	2.4 (0.9)	0.952	2.4 (1.1)	2.5 (1.0)	0.624	2.3 (0.9)	2.4 (0.9)	0.545
Systolic blood pressure on admission, mmHg	133.0 (20.5)	128.8 (21.5)	0.000	127.1 (20.1)	121.3 (21.4)	0.020	129.7 (20.6)	124.5 (21.5)	0.036	134.2 (20.3)	131.5 (20.9)	0.006
Diastolic blood pressure on admission, mmHg	77.2 (11.9)	75.9 (12.5)	0.009	76.7 (11.5)	75.4 (14.1)	0.400	76.7 (12.2)	75.5 (13.1)	0.457	77.3 (12.0)	76.1 (12.0)	0.029
Patients with stent implantation, *n* (%)	702 (60.9)	906 (72.4)	0.000	64 (55.1)	126 (69.6)	0.011	63 (55.3)	171 (78.1)	0.000	571 (62.0)	600 (70.5)	0.000
Number of stents	1.2 (1.3)	1.3 (1.2)	0.139	1.13 (1.32)	1.30 (1.30)	0.253	1.2 (1.4)	1.3 (1.1)	0.334	1.2 (1.3)	1.3 (1.2)	0.489
Number of lesions												
One vessel, *n* (%)	481 (41.8)	580 (46.4)	0.021	36 (30.5)	70 (38.7)	0.149	41 (36.0)	101 (46.1)	0.075	404 (43.9)	409 (48.2)	0.072
Two vessels, *n* (%)	276 (24.0)	329 (26.3)	0.179	31 (26.3)	46 (25.4)	0.868	26 (22.8)	59 (26.9)	0.412	219 (23.8)	224 (26.4)	0.211
Three vessels, *n* (%)	273 (23.7)	243 (19.5)	0.011	40 (33.9)	46 (25.4)	0.113	37 (32.5)	39 (17.8)	0.003	196 (21.3)	158 (18.6)	0.157
Left main artery, *n* (%)	122 (10.6)	97 (7.8)	0.016	11 (9.3)	19 (10.5)	0.741	10 (8.8)	20 (9.1)	0.913	101 (11.0)	58 (6.8)	0.002
Medicines before admission												
Aspirin, *n* (%)	493 (42.8)	439 (35.1)	0.000	49 (41.5)	56 (30.9)	0.061	39 (34.2)	54 (24.7)	0.065	405 (44.0)	329 (38.7)	0.023
Clopedogrel, *n* (%)	254 (22.0)	260 (20.8)	0.457	25 (21.2)	37 (20.4)	0.877	18 (15.8)	33 (15.1)	0.862	211 (22.9)	190 (22.3)	0.769
β-Blockers, *n* (%)	344 (29.8)	325 (26.0)	0.035	31 (26.3)	42 (23.2)	0.546	22 (19.3)	26 (11.9)	0.067	291 (31.6)	257 (30.2)	0.525
Statin, *n* (%)	320 (27.8)	293 (23.4)	0.015	32 (27.1)	37 (20.4)	0.180	22 (19.3)	25 (11.4)	0.050	266 (28.9)	231 (27.1)	0.416
ACEI/ARB, *n* (%)	285 (24.7)	252 (20.1)	0.007	23 (19.5)	29 (16.0)	0.439	26 (22.8)	25 (11.4)	0.006	236 (25.6)	198 (23.3)	0.249
CCB, *n* (%)	325 (28.2)	249 (19.9)	0.000	20 (16.9)	23 (12.7)	0.307	24 (21.1)	21 (9.6)	0.004	281 (30.5)	205 (24.1)	0.002
Medicines at discharge												
Aspirin, *n* (%)	1,097 (95.1)	1,187 (94.9)	0.771	107 (90.7)	172 (95.0)	0.141	108 (94.7)	209 (95.4)	0.778	882 (95.8)	806 (94.7)	0.297
Clopedogrel, *n* (%)	1,065 (92.4)	1,146 (91.6)	0.493	108 (91.5)	167 (92.3)	0.818	105 (92.1)	204 (93.2)	0.726	852 (92.5)	775 (91.1)	0.270
Statin, *n* (%)	1,029 (89.2)	1,190 (95.1)	0.000	109 (92.4)	174 (96.1)	0.158	99 (86.8)	206 (94.1)	0.024	821 (89.1)	810 (95.2)	0.000
CCB, *n* (%)	375 (32.5)	305 (24.4)	0.000	20 (16.9)	19 (10.5)	0.105	26 (22.8)	28 (12.8)	0.019	329 (35.7)	258 (30.3)	0.016
ACEI/ARB, *n* (%)	675 (58.5)	749 (59.9)	0.508	73 (61.9)	97 (53.6)	0.158	67 (58.8)	134 (61.2)	0.669	535 (58.1)	518 (60.9)	0.234
β-Blocker, *n* (%)	821 (71.2)	838 (67.0)	0.025	85 (72)	115 (63.5)	0.127	75 (65.8)	152 (69.4)	0.501	661 (71.8)	571 (67.1)	0.033

LVEF, left ventricular ejection fraction; ACS, acute coronary syndrome; MI, myocardial infarction; PCI, percutaneous coronary intervention; BMI, body mass index; HDL-C, high density lipoprotein cholesterol; LDL-C, low density lipoprotein cholesterol; ACEI, angiotensin-converting enzyme inhibitors; ARB, angiotensin receptor blocker; CCB, calcium channel blocker.

### 3.1 Endpoints

#### 3.1.1 Total patients

**Figure 2:**
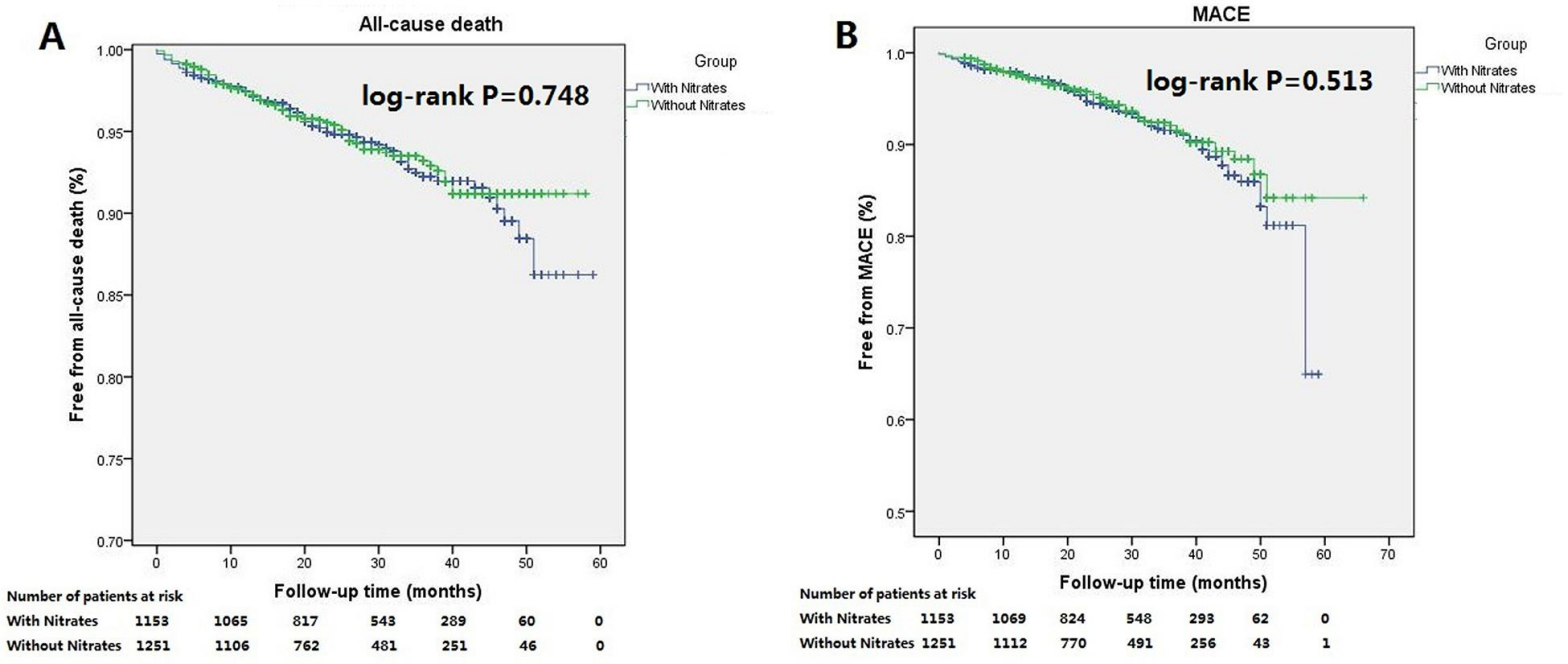
Kaplan–Meier curves for the all-cause death and MACE in the total patient population stratiﬁed according to discharged or not discharged on nitrates. There were no statistically signiﬁcant difference in cumulative incidences of all-cause death (**A**, log-rank *P* = 0.748) and MACE (**B**, log-rank *P* = 0.513) between patients discharged on nitrates and not discharged on nitrates.

Among patients with nitrates at discharge, 72 patients (6.2%) died during follow-up, compared with 67 (5.4%) deaths among patients without nitrates. The cumulative incidence for all-cause death was insignificant between patients with nitrates and without nitrates at discharge (log-rank *P* = 0.748; Figure 2A). Similarly, no difference in cumulative incidence for MACE was observed (log-rank *p* = 0.513; Figure 2B). Multivariate Cox regression analysis showed that discharged on nitrates was not associated with the occurrence of all-cause death or MACE among the overall cohort (Table 2). Age, LVEF, serum creatinine, white blood cell, taking β-blocker before admission, prescribed aspirin at discharge, and stent implantation were independent predictors of all-cause death. LVEF, β-blocker use before admission, aspirin prescription at discharge, stent implantation, and left main artery disease were independently associated with MACE in these patients (Table 2).

**Table 2:** Multivariate cox regression analysis to assess independent correlates of all-cause death and MACE in the total patient population.

Variates	All-cause death			MACE		
	HR	95% CI	*P*	HR	95% CI	*P*
Discharged on nitrates	1.08	0.75–1.54	0.695	1.20	0.87–1.66	0.269
Age	1.07	1.05–1.10	0.000	1.02	1.00–1.03	0.114
LVEF	0.96	0.95–0.98	0.000	0.96	0.95–0.98	0.000
Serum creatinine	1.00	1.00–1.01	0.000	1.00	1.00–1.00	0.090
White blood cell	1.09	1.03–1.15	0.005	1.05	0.99–1.10	0.098
Stent implantation	0.56	0.38–0.81	0.002	0.59	0.42–0.82	0.002
Left main artery disease	1.48	0.80–2.74	0.220	1.87	1.12–3.14	0.017
Medicines before admission						
β-Blocker	0.59	0.36–0.97	0.036	0.61	0.39–0.95	0.029
Medicines at discharge						
Aspirin	0.43	0.25–0.74	0.002	0.54	0.29–0.98	0.043

The Enter method was used. Only variable with a *P* ≤ 0.05 was shown except for discharged on nitrates. Adjusted factors consisted of discharged on nitrates, age, male, history of hypertension, diabetes mellitus, MI, current smoking, BMI, LVEF, serum creatinine, blood glucose, white blood cell, systolic blood pressure on admission, diastolic blood pressure on admission, stent implantation, one-vessel disease, three-vessel disease, left main artery disease, and medicines before admission and at discharge.

LVEF, left ventricular ejection fraction; HR, hazard ratio; CI, confidence interval; MACE, major adverse cardiovascular events.

#### 3.1.2 Patients with LVEF < 45%

##### 3.1.2.1 Discharge on nitrates and all-cause death

**Figure 3:**
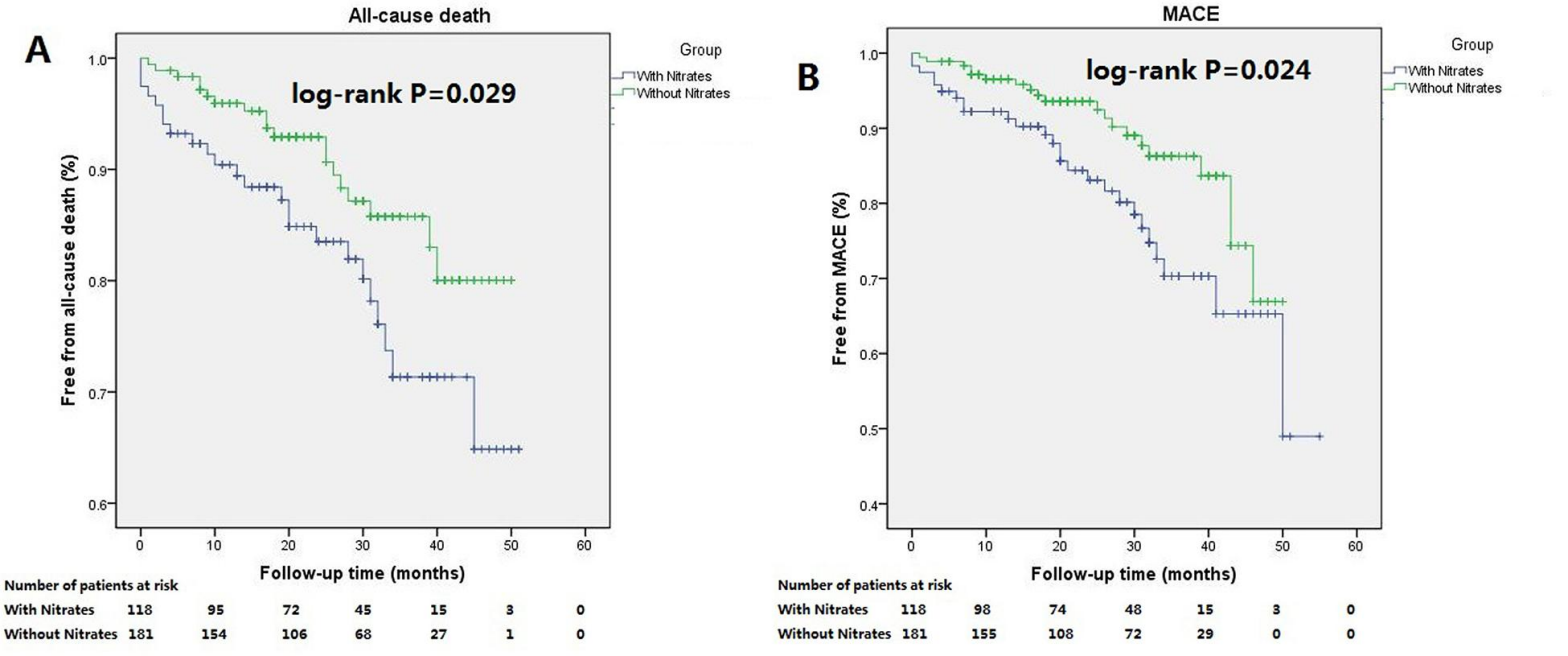
Kaplan–Meier curves for the all-cause death and MACE in patients with LVEF less than 45% stratiﬁed according to discharged or not discharged on nitrates. Patients discharged on nitrates had statistically signiﬁcant worse outcomes with higher cumulative incidence of all-cause death (**A**, log-rank *P* = 0.029), and MACE (**B**, log-rank *P* = 0.024).

During the follow-up, 43 patients (14.3%) died. Of these, 24 deaths occurred among patients with nitrates at discharge, and 19 deaths were among those without nitrates. The cumulative incidence of all-cause death was higher in patients with nitrates at discharge (log-rank *P* = 0.029; Figure 3A). Multivariate Cox regression analysis identified discharged on nitrates, age, current smoking, and serum creatinine as independent predictors of all-cause death. Discharge on nitrates was associated with an HR of 2.14 (95% CI, 1.15–3.96) after adjusting for confounding factors (Table 3).

**Table 3:** Multivariate cox regression analysis to assess independent correlates of all-cause death and MACE in patients with LVEF less than 45%.

	All-cause death				MACE		
Variates	HR	95% CI	*P*	Variates	HR	95% CI	*P*
Discharged on nitrates	2.14	1.15–3.96	0.016	Discharged on nitrates	1.91	1.07–3.43	0.03
Age	1.06	1.02–1.10	0.001				
Current smoking	2.37	1.16–4.83	0.018				
Serum Creatinine	1.01	1.01–1.02	0.000				

The Forward method was used. Adjusted for discharged on nitrates, body mass index, age, male, history of hypertension, diabetes, current smoking, serum creatinine, aspirin, clopidogrel, statin and β-blocker at discharge, systolic blood pressure on admission, and stent implantation.

HR, hazard ratio; CI, confidence interval; MACE, major adverse cardiovascular events.

##### 3.1.2.2 Discharge on nitrates and MACE

MACE was reached in 44 patients (14.7%). Increased cumulative incidence of MACE was observed in patients with nitrates at discharge (log-rank *p* = 0.024, Figure 3B). However, after adjusting for confounding factors, the Multivariate Cox regression analysis showed that discharge on nitrates was the only independent predictor of MACE, with an HR of 1.91 (95% CI, 1.07–3.43) (Table 3).

#### 3.1.3 Patients with LVEF between 45% and 55%

**Figure 4:**
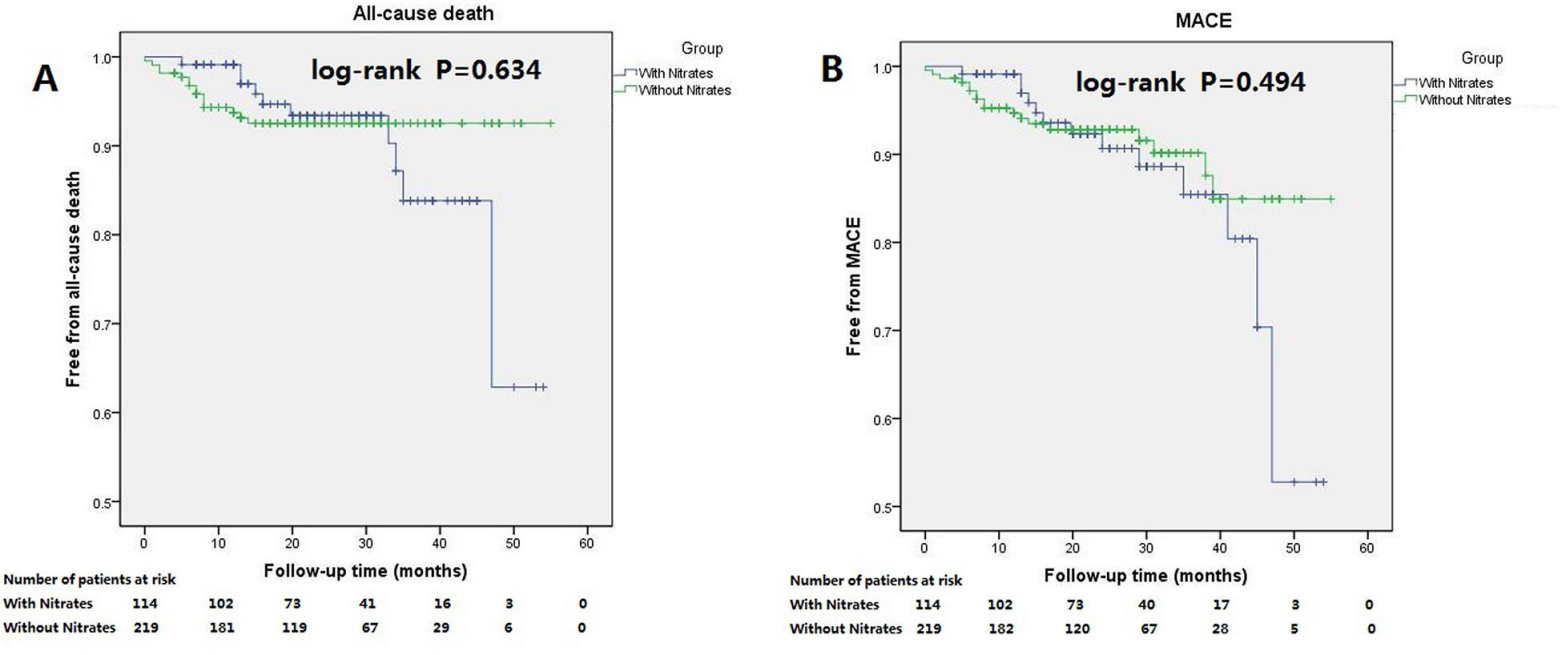
Kaplan–Meier curves for the all-cause death and MACE in patients with LVEF between 45% and 55% stratiﬁed according to discharged or not discharged on nitrates. Patients discharged or not discharged on nitrates had similar long-term outcome showing insignificantly difference in cumulative incidences of all-cause death (**A**, log-rank *P* = 0.634), and MACE (**B**, log-rank *P* = 0.494).

Among patients with LVEF between 45% and 55%, discharge on nitrates was not associated with all-cause death or MACE. Compared with patients not discharged on nitrates, those with discharged on nitrates had similar cumulative incidences of all-cause death and MACE (Figure 4. A: all-cause death, log-rank *P* = 0.634; B: MACE, log-rank *P* = 0.494). Multivariate Cox regression analysis also showed that discharge on nitrates was not an independent predictor of all-cause death or MACE (Table 4). Age, serum creatinine, and aspirin prescription at discharge were independent predictors of all-cause death. Body mass index and serum creatinine predicted MACE independently in patients with LVEF between 45% and 55%.

**Table 4:** Multivariate cox regression analysis to assess independent correlates of all-cause death and MACE in patients with LVEF between 45% and 55%.

Variates	All-cause death			MACE		
	HR	95% CI	*P*	HR	95% CI	*P*
Discharged on nitrates	0.63	0.20–1.96	0.426	1.10	0.42–2.88	0.853
Age	1.11	1.02–1.20	0.017	1.07	1.01–1.14	0.025
Serum creatinine	1.01	1.00–1.01	0.003	1.00	1.00–1.01	0.034
Medicines at discharge						
Aspirin	0.04	0.01–0.22	0.000	0.16	0.03–0.80	0.026

The Enter method was used. Only variable with a *P* ≤ 0.05 was shown except for discharged on nitrates. Adjusted factors consisted of discharged on nitrates, age, male, history of hypertension, diabetes mellitus, MI, current smoking, BMI, serum creatinine, LDL-C, systolic blood pressure on admission, stent implantation, one-vessel disease, three-vessel disease, left main artery disease, and medicines before admission and at discharge.

HR, hazard ratio; CI, confidence interval; MACE, major adverse cardiovascular events.

#### 3.1.4 Patients with LVEF more than 55%

**Figure 5:**
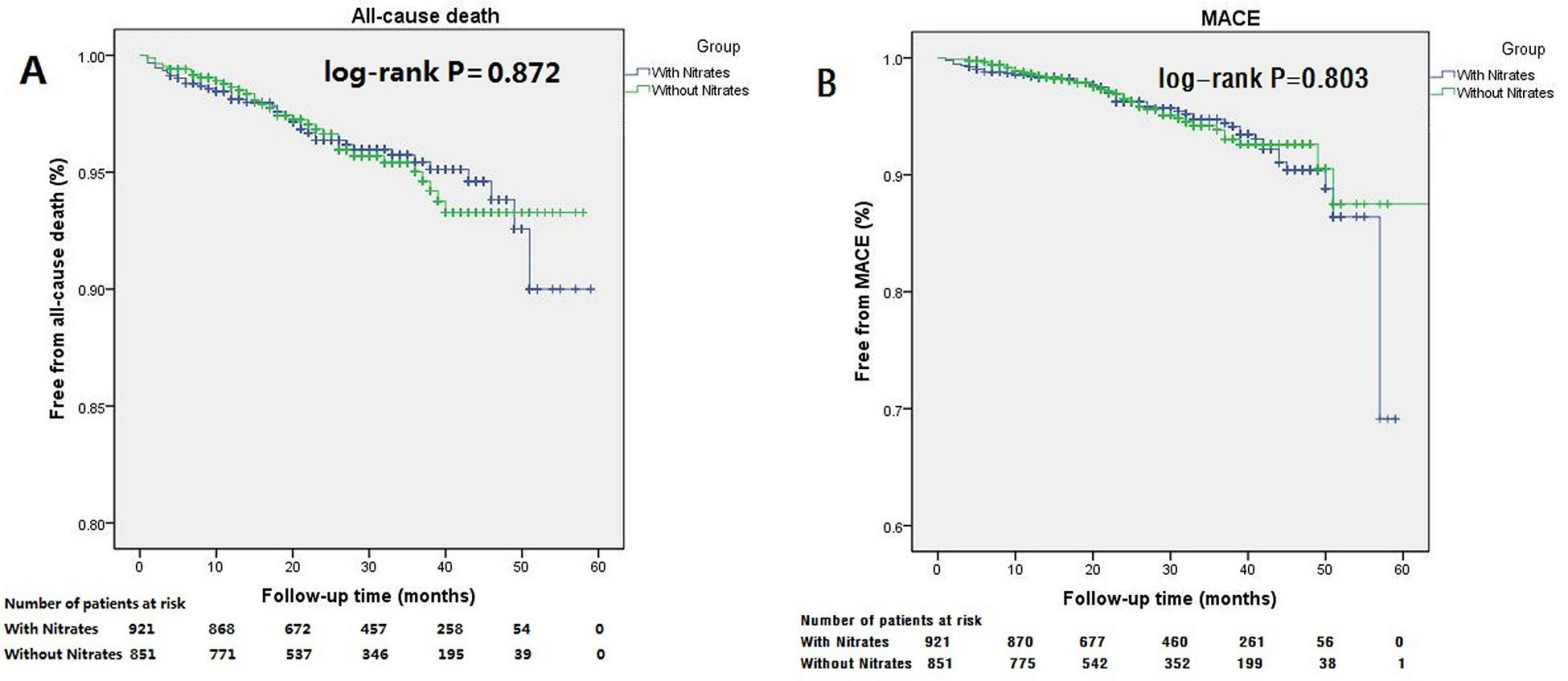
Kaplan–Meier curves for the all-cause death and MACE in patients with LVEF more than 55% stratiﬁed according to discharged or not discharged on nitrates. Patients discharged or not discharged on nitrates had similar long-term outcome showing insignificantly difference in cumulative incidences of all-cause death (**A**, log-rank *P* = 0.872), and MACE (**B**, log-rank *P* = 0.803).

There were 38 all-cause deaths and 49 MACEs among patients with nitrates at discharge. For patients without nitrates at discharge, 33 died and 38 reached MACE. The differences of cumulative incidence for all-cause death and MACE between patients with nitrates and without nitrates at discharge were both insignificant (Figure 5A: all-cause death, log-rank *P* = 0.872; Figure 5B: MACE, log-rank *P* = 0.803). Similarly, multivariable Cox regression analysis also showed that discharge on nitrates was not an independent predictor of all-cause death or MACE. Age, LDL-C, and white blood cell were independently associated with all-cause death. Left main artery disease, number of stents, stent implantation, and statin prescription at discharge were independent predictors of MACE (Table 5).

**Table 5:** Multivariate cox regression analysis to assess independent correlates of all-cause death and MACE in patients with LVEF more than 55%.

Variate	All-cause death			MACE		
	HR	95% CI	*P*	HR	95% CI	*P*
Discharged on nitrates	0.83	0.49–1.41	0.495	0.97	0.60–1.56	0.891
Age	1.09	1.05–1.13	0.000	1.00	0.97–1.02	0.790
Serum creatinine	1.00	1.00–1.01	0.002	1.00	1.00–1.01	0.166
White blood cell	1.15	1.06–1.25	0.001	1.06	0.97–1.15	0.193
Stent implantation	0.50	0.28–0.88	0.015	0.53	0.32–0.88	0.014
Left main artery disease	2.25	0.96–5.27	0.063	3.07	1.51–6.23	0.002
Medicines at discharge						
Statin	0.79	0.35–1.75	0.559	4.20	1.25–14.16	0.02

The Enter method was used. Only variable with a *P* ≤ 0.05 was shown except for discharged on nitrates. Adjusted factors consisted of discharged on nitrates, age, male, history of hypertension, diabetes mellitus, MI, current smoking, LVEF, serum creatinine, LDL-C, white blood cell, systolic blood pressure on admission, diastolic blood pressure on admission, stent implantation, one-vessel disease, three-vessel disease, left main artery disease, and medicines before admission and at discharge.

HR, hazard ratio; CI, confidence interval; MACE, major adverse cardiovascular events.

### 3.2 Reclassified groups

Reclassification followed the ESC heart failure nomenclature (≤40%, 41%–49%, and ≥50%) (16) to ensure comparability with current guideline categories. Considering that some guidelines and studies classify LVEF into normal, mildly abnormal, and moderately and severely abnormal with LVEF ≥ 50%, 40%–50%, and ≤40%, respectively (3), we reclassified patients into the following three groups: LVEF ≥ 50%, 40%–50%, and ≤40%. There were 206 patients with LVEF ≤ 40%, 234 with LVEF between 40% and 50%, and 1964 with LVEF ≥ 50%. The baseline differences between patients discharged on nitrates and those not on nitrates were comparable in each subgroup compared with the former grouping strategy. Similarly, the reclassified strategy also noted that discharge on nitrates was an independent predictor factor of MACE and all-cause death in patients with LVEF ≤ 40%, but had no association with MACE or all-cause death in patients with LVEF between 40% and 50% or LVEF ≥ 50% (data not shown).

## 4 Discussion

Chronic nitrate therapy remains widely used for symptom control in CAD, yet its long-term prognostic impact has not been established in contemporary practice. Moreover, whether baseline left ventricular systolic function modifies any association between nitrates and clinical outcomes has been insufficiently characterized. In our angiography-confirmed CAD cohort, nearly half of the patients were discharged on long-acting nitrates. In the overall population, nitrate prescription at discharge was not associated with all-cause mortality or MACE. However, after stratification by LVEF, an adverse association was confined to patients with LVEF < 45%, in whom discharge on nitrates was independently associated with higher risks of all-cause death and MACE; no significant associations were observed in patients with LVEF 45%–55% or >55%.

These findings are clinically relevant because long-acting nitrates continue to be prescribed frequently in coronary artery disease, despite limited evidence for sustained prognostic benefit. Current guidelines recommend long-acting nitrates primarily for angina prevention and symptom relief, particularly when symptoms persist despite first-line antianginal therapy (13). In contrast, several observational studies have raised concerns about chronic nitrate exposure in ischemic heart disease. Using databases from two large postinfarction studies, Nakamura and colleagues reported an association between long-term nitrate use and higher mortality (17, 18). Similarly, Ishikawa et al. found that long-term oral nitrate therapy was associated with more cardiac events in a cohort of patients with healed myocardial infarction (4), and Kanamasa et al. reported higher event rates during follow-up among patients treated continuously with oral and transdermal nitrates (6). More contemporary registry data have extended these observations to other clinical contexts. For example, among higher-risk stable coronary disease populations—including patients with diabetes undergoing elective percutaneous coronary intervention—chronic oral nitrate therapy has been linked to increased major adverse cardiovascular events in observational analyses (19). In addition, in cohorts of vasospastic angina, long-acting nitrates have not demonstrated prognostic benefit, and certain nitrate-based regimens have been associated with higher rates of adverse events (20–22). Taken together, although these data are subject to confounding by indication, the consistency of signals across diverse cohorts supports the possibility that chronic nitrate therapy may be prognostically non-neutral in selected subgroups, which provides clinical context for our finding of an adverse association primarily in patients with reduced LVEF.

However, most prior studies have not evaluated whether baseline LVEF modifies the association between chronic nitrate therapy and clinical outcomes in coronary artery disease. To address this gap, we assessed discharge prescriptions for long-acting nitrates across prespecified LVEF strata in an angiographically confirmed CAD cohort. The primary finding was that an adverse association with both all-cause mortality and major adverse cardiovascular events was observed among patients with LVEF < 45%, whereas no significant associations were detected among those with LVEF ≥ 45%. Although the observational design precludes causal inference and residual confounding remains possible, these data suggest that the clinical risk profile associated with chronic nitrate therapy may differ according to baseline systolic function.

Although these results may appear to conflict with heart failure trials that evaluated nitrate-based regimens, several distinctions are important. First, the trials most often cited in support of nitrate therapy in HFrEF evaluated hydralazine plus isosorbide dinitrate rather than nitrate monotherapy, and they were conducted in therapeutic eras that differed substantially from contemporary guideline-directed medical therapy (8, 23). In our cohort, most patients received background neurohormonal therapies, including an ACEI or ARB and a beta blocker, which may influence both symptoms and prognosis and may also modify the net effect of vasodilator therapy. In A HeFT, which enrolled African American patients with heart failure, hydralazine plus isosorbide dinitrate was associated with reduced mortality; however, the restriction to a single racial group and early trial termination limit generalizability, particularly to non-Black populations (8). Consistently, a large observational cohort study including 76,828 patients with heart failure did not show an apparent benefit of hydralazine plus nitrate therapy overall or within major racial or ethnic subgroups (24). Furthermore, contemporary guidance positions hydralazine plus isosorbide dinitrate as a therapy for selected patients with HFrEF rather than a broadly applicable nitrate strategy to improve prognosis (25). Finally, more recent randomized data in HFpEF have not supported benefit from nitrate-based approaches. Extended release isosorbide mononitrate reduced daily activity without improving exercise capacity or quality of life in NEAT HFpEF (26), and inhaled inorganic nitrite did not improve peak oxygen consumption in INDIE HFpEF (27). Taken together, the broader contemporary literature suggests that nitrate-related strategies are not uniformly beneficial across heart failure phenotypes, which is compatible with our observation that adverse associations were confined to CAD patients with reduced LVEF.

Nitrate tolerance, the potential role of intermittent nitrate therapy, neurohumoral effects, aggravation of myocardial ischemia, and hypervascular responses in coronary arteries may contribute to the adverse outcomes associated with chronic nitrate therapy (28, 29). In the setting of prior myocardial infarction, the ischemic myocardial microenvironment is often characterized by persistent oxidative stress, inflammation, and microvascular dysfunction, which may further impair myocardial and vascular homeostasis (30). In addition, the thrombotic microenvironment in postmyocardial infarction patients can exacerbate endothelial dysfunction, further compromising vascular integrity and increasing the risk of adverse cardiovascular events (31, 32). These factors may contribute to the impaired endothelial function observed in patients with reduced LVEF, which could amplify their vulnerability to additional endothelial or hemodynamic stress. As a result, this adverse ischemic milieu may potentially contribute to the unfavorable associations observed with chronic nitrate therapy in these patients. In addition, both experimental and human studies have suggested that sustained exposure to organic nitrates may impair endothelial function through oxidative stress-related pathways (33, 34). In a well-characterized animal model, short-term treatment with isosorbide 5 mononitrate was associated with endothelial dysfunction and increased oxidative stress even in the absence of clear hemodynamic tolerance, which supports the possibility that vascular dysfunction may occur independently of classic tolerance (33). Consistently, once-daily isosorbide 5 mononitrate therapy impaired endothelial-dependent vasomotor function in humans, and this effect was reversed by antioxidant supplementation, further implicating reactive oxygen species in this process (34). Because hydralazine has been reported to exert antioxidant-related effects, it is possible that concomitant hydralazine could attenuate nitrate-associated oxidative stress, thereby influencing the net clinical effects of combination therapy; however, this remains a mechanistic hypothesis rather than a definitive explanation for trial outcomes (35). Endothelial dysfunction is an established marker of worse long-term prognosis in patients with coronary artery disease and heart failure (36–38). Moreover, patients with reduced systolic function may have less physiologic reserve and could be more vulnerable to additional vascular dysfunction, which may help explain why adverse associations with nitrates were observed in patients with LVEF < 45% but not in those with preserved systolic function (37–39).

In addition to the primary exposure of interest, several established clinical and treatment-related factors were independently associated with outcomes, supporting the overall clinical plausibility of our models. In the Cox regression analyses, preadmission beta blocker use, higher LVEF, discharge aspirin prescription, and stent implantation were associated with lower risks of all-cause mortality and major adverse cardiovascular events, whereas older age, higher serum creatinine and white blood cell count, left main coronary artery disease, and current smoking were associated with higher risks of these outcomes. These associations are consistent with prior reports in patients with coronary artery disease and heart failure (40–44). By contrast, the direction of the association between discharge statin therapy and outcomes in the subgroup with LVEF greater than 55% did not align with the broader literature supporting statins for secondary prevention (13, 45). This finding should be interpreted cautiously because only a small proportion of patients were not discharged on statins, which may have limited statistical power and increased susceptibility to imbalance and model instability. In addition, residual confounding and treatment selection related to disease severity or comorbidity could have influenced this estimate. Accordingly, while these covariate associations provide clinical context for our main findings, the statin signal in this subgroup should be viewed as exploratory and is further addressed in the limitations.

## 5 Limitations

This study has several limitations. First, its retrospective, single-center observational design precludes causal inference and leaves the possibility of residual confounding despite multivariable adjustment. Second, nitrate exposure was defined according to discharge prescription, and detailed information on postdischarge adherence, treatment duration, or dose changes was not available, which may have resulted in exposure misclassification. Third, coronary artery disease was defined using angiographic criteria without routine physiological assessment, and anatomical stenosis severity may not always reflect functional ischemic significance. Fourth, echocardiographic data were not available for all patients undergoing coronary angiography, potentially introducing selection bias. Finally, as the cohort was derived from a single center, the generalizability of these findings to other populations requires external validation.

## 6 Conclusion

Our study suggests that nearly half of patients with coronary artery disease are discharged on nitrates. The impact of prescribed nitrates at discharge on prognosis is diverse across LVEF. Being discharged on nitrates was not a predictor of MACE or all-cause death in patients with normal or mildly reduced LVEF, but it was a strong predictor among those with moderately or severely reduced LVEF.

## Data Availability

The raw data supporting the conclusions of this article will be made available by the authors, without undue reservation.
